# At the Intersection of Major and Minor Spliceosomes: Crosstalk Mechanisms and Their Impact on Gene Expression

**DOI:** 10.3389/fgene.2021.700744

**Published:** 2021-07-20

**Authors:** Maureen V. Akinyi, Mikko J. Frilander

**Affiliations:** Institute of Biotechnology/Helsinki Institute of Life Science, University of Helsinki, Helsinki, Finland

**Keywords:** RNA processing, mRNA splicing, minor spliceosome, major spliceosome, exon definition, cryptic splicing, minor spliceosome disease

## Abstract

Many eukaryotic species contain two separate molecular machineries for removing non-coding intron sequences from pre-mRNA molecules. The majority of introns (more than 99.5% in humans) are recognized and excised by the major spliceosome, which utilizes relatively poorly conserved sequence elements at the 5′ and 3′ ends of the intron that are used for intron recognition and in subsequent catalysis. In contrast, the minor spliceosome targets a rare group of introns (approximately 0.5% in humans) with highly conserved sequences at the 5′ and 3′ ends of the intron. Minor introns coexist in the same genes with major introns and while the two intron types are spliced by separate spliceosomes, the two splicing machineries can interact with one another to shape mRNA processing events in genes containing minor introns. Here, we review known cooperative and competitive interactions between the two spliceosomes and discuss the mechanistic basis of the spliceosome crosstalk, its regulatory significance, and impact on spliceosome diseases.

## Introduction

The removal on non-coding intervening sequences (introns) and ligation of coding sequences (exons) in pre-cursor messenger RNA (pre-mRNA), is carried out by a dynamic and complex machinery known as the spliceosome. The majority of metazoan organisms contain two parallel but analogous spliceosomes: the major or U2-dependent spliceosome which excises approximately 99.5% of introns, depending on the organism, and the minor or U12-dependent spliceosome which excises about 0.5% of introns. The respective intron types are similarly referred to as either major or U2-type introns, and minor or U12-type introns (Turunen et al., 2013a). The number of U12-type introns varies between species: for instance, in humans approximately 700 genes contain U12-type introns, while only 19 are found in *Drosophila*. More recently, an investigation of the genome of slime mold *Physarum polycephalum* revealed an exceptional case of >20,000 minor introns in a single genome (Larue et al., 2021). The typical architecture of minor intron containing genes (MIGs) includes a single U12-type intron per gene, flanked by multiple U2-type introns. However, a small subset of MIGs contain two or even three U12-type introns (Burge et al., 1998; Levine and Durbin, 2001; Moyer et al., 2020). The origin of the two parallel machineries and the disproportionate distribution of the two intron types in present day genomes has been the subject of ongoing debate (Burge et al., 1998; Lynch and Richardson, 2002; Roy and Gilbert, 2006; Lin et al., 2010; Baumgartner et al., 2019). Nonetheless, it is safe to conclude that both machineries are ancient, related to one another, and originate from group II self-splicing introns (Burge et al., 1998; Shi, 2017). Interestingly, U12-type introns have reportedly been lost in multiple phylogenetic lineages, suggesting that they can be dispensable (Bartschat and Samuelsson, 2010). On the other hand, recognition sequences and locations of U12-type introns in individual genes are highly conserved in organismal lineages that have retained them (Moyer et al., 2020). These properties suggest that U12-type introns may serve an indispensable regulatory function in present-day organisms. Intriguingly, some introns harbor either tandem or overlapping splice sites that enable intron recognition by both major and minor spliceosomes, which in some cases, has been shown to have regulatory significance (Scamborova et al., 2004; Janice et al., 2013; Hafez and Hausner, 2015).

**FIGURE 1:**
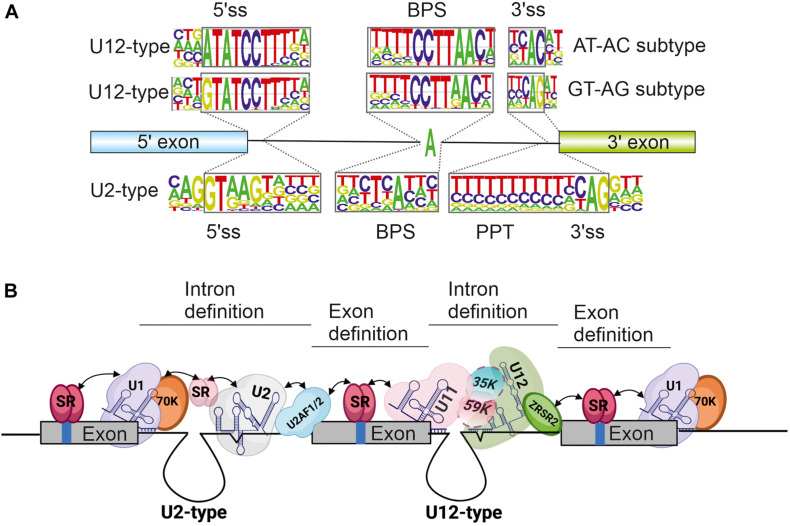
**(A)** Consensus splice site sequences of minor and major introns. Minor or U12-type introns can additionally be grouped into AT-AC and GT-AG subtypes based on the first and last di-nucleotides. **(B)** Schematic of exon definition interactions taking place in a typical minor intron containing gene flanked by U2-type introns. Selected snRNA and protein components are indicated. Exons are depicted as solid boxes and introns as lines. Regulatory elements within exons are depicted as blue bars. Figure in panel **(B)** was modified from Turunen et al. (2013a). Created by BioRender.com.

A key distinguishing feature between U12-type and U2-type introns is the conservation of intron recognition sequences i.e., the 5′ splice site (5′ss), 3′ splice site (3′ss) and the branch point sequence (BPS). Additionally, U2-type introns distinctively contain a polypyrimidine tract (PPT) upstream of the 3′ss. Splice site sequences are relatively weakly conserved in U2-type introns, which leads to more flexible splice site choices that fuel alternative splicing processes. In contrast, splice site sequences within U12-type introns are significantly more conserved (Figure 1A), which translates to less flexibility in splice site choice and consequently, reduced levels of alternative splicing in minor introns. Despite these differences, the overall splicing chemistry and spliceosome assembly is similar between the two intron types and has been covered in depth in several reviews (Patel and Steitz, 2003; Singh and Cooper, 2012; Turunen et al., 2013a; Matera and Wang, 2014; Jutzi et al., 2018; Wilkinson et al., 2020). Briefly, the 5′ss and BPS are initially recognized either by separate U1 and U2 snRNPs (major spliceosome) or a U11/U12 di-snRNP complex (minor spliceosome). Additionally, the PPT and 3′ss in U2-type introns are recognized by the U2AF1/U2AF2 protein heterodimer, whereas the U12-type intron 3′ss is recognized by the ZRSR2 protein. Following this initial recognition, the entry of U4/U6.U5 tri-snRNP (major spliceosome) or U4atac/U6atac.U5 tri-snRNP (minor spliceosome) leads to the formation of catalytic structures and intron excision.

Efforts aimed at understanding the functional significance of U12-type introns have suggested that the splicing of minor introns is slower or less efficient than that of major introns and could be used as a rate-limiting step to control or fine-tune mRNA levels of MIGs (Patel et al., 2002; Younis et al., 2013; Niemelä et al., 2014). Accordingly, several studies have reported elevated levels of retained U12-type introns under physiological conditions and in human diseases caused by mutations in minor spliceosome components. The retention of a single U12-type intron can disrupt the reading frame which would likely lead to downregulation at the protein level, either through nuclear retention and degradation of mRNAs containing unspliced U12-type introns (Niemelä et al., 2014; Ogami et al., 2018; Palazzo and Lee, 2018) or nonsense-mediated decay (NMD) due to introduction of premature termination codons (PTC) (Kurosaki et al., 2019). Consequently, studies examining the regulatory significance of U12-type introns or minor spliceosome diseases have mostly focused on intron retention, which is typically the predominant outcome of regulated or defective U12-type intron splicing. In this review, we instead focus on the mechanisms that involve interplay between adjacent minor and major spliceosomes during nuclear mRNA processing. We review mechanisms of minor and major spliceosome interactions, their potential regulatory significance under physiological conditions and their impact on minor spliceosome diseases.

## Cooperation and Competition in Recognition of Adjacent Splice Sites as a Means of Regulation

Interaction between the minor and major spliceosomes during pre-mRNA processing can lead to two opposing outcomes: cooperation or competition. Cooperative interactions can facilitate mutual activation of adjacent spliceosomes during splicing through exon and intron definition mechanisms. Alternatively, the two spliceosomes can compete with one another for access to introns that harbor splice site recognition sequences for both machineries. Under normal physiological conditions, competitive interactions can be identified by the alternate use of either U12-type or U2-type splice sites, which result in different mRNA isoforms. In contrast, cooperative interactions between the two spliceosomes are more challenging to identify, as they typically do not lead to changes in alternative splice site usage under physiological conditions, except in the few cases where such interactions have been exploited for regulatory purposes. Additionally, diseases affecting minor spliceosome functions lead to a variety of alternative splicing choices in both interaction types.

### Cooperation Between the Minor and Major Spliceosomes

At the mechanistic level, mutual interactions between the major and minor spliceosomes on the same pre-mRNA are predominantly mediated by exon definition interactions, in which the initial recognition of introns takes place by pairing splice sites across exons instead of introns (Robberson et al., 1990; Berget, 1995). Subsequently, the juxtaposition of exon-definition complexes enables the cross-intron pairing of splice sites through protein-protein interactions. Exon definition mechanisms are particularly useful in describing intron recognition mechanisms in vertebrates, which have the characteristic pre-mRNA architecture of relatively short exons separated by long introns. In contrast, the recognition of short introns occurs through intron-definition mechanisms whereby initial splice site pairing takes place across introns (Berget, 1995).

**FIGURE 2:**
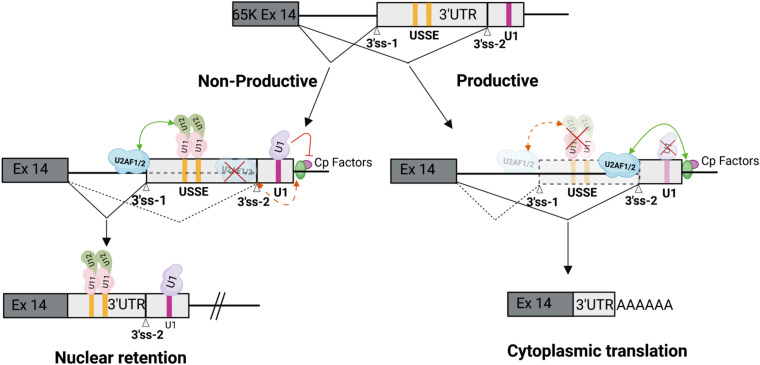
Cooperation between minor and major spliceosomes. Feedback regulation in the minor *RNPC3*(65K) gene is mediated by protein-protein interactions between the U11/U12 di-snRNP bound to the USSE and upstream U2AF1/2 to enhance recognition of an upstream 3′ss. Cp factors denote cleavage and polyadenylation factors. This leads to formation of either productive mRNA translated in the cytoplasm, or unproductive mRNA that is retained in the nucleus. Modified from Verbeeren et al. (2017). Created by BioRender.com.

Both exon and intron definition mechanisms rely on protein-protein interactions to connect spliceosomal complexes assembled on the 5′ss or PPT/3′ss, to enable cross-exon and cross-intron communication. Proteins containing arginine and serine rich domains (RS domains) are the main facilitators of these interactions (Figure 1B). These include the SR protein family of splicing regulators and several integral spliceosome components present in both the major and minor spliceosomes (Long and Caceres, 2009). The latter group includes the U1-70K protein that is part of the U1 snRNP in the major spliceosome (Theissen et al., 1986; Spritz et al., 1987; Cho et al., 2011), its paralog U11-35K in the minor spliceosome which is also a component of the U11 snRNP (Will et al., 2004; Niemelä et al., 2015), the U2AF1/2 heterodimer involved in initial recognition of the PPT and 3′ss of U2-type introns, and the ZRSR2 protein that functions in recognition of the U12-type 3′ss (Tronchère et al., 1997; Shen et al., 2010). Additionally, recent work examining exon-bridging disruptions between major and minor spliceosomes provided evidence for a role of the U11-59K protein in exon-definition interactions (Olthof et al., 2021). Further regulation of both exon and intron definition mechanisms is provided by embedded exonic and intronic sequence elements, that are bound by RNA binding proteins (RBPs) including SR-proteins and heterogenous ribonucleoproteins (hnRNPs). Both SR and hnRNP proteins predominantly facilitate and regulate recognition of major introns (Berget, 1995; Reed, 1996; De Conti et al., 2013), but have also been shown to similarly interact with the minor spliceosome (Hastings and Krainer, 2001; Shen and Green, 2007; Turunen et al., 2013b). Consistently, exon-definition interactions between the major and minor spliceosomes have been demonstrated both *in vitro*, between U1 snRNP and the minor spliceosome (Wu and Krainer, 1996), and *in vivo*, between U11/U12 di-snRNP and the upstream U2AF1/2 bound to the major spliceosome PPT and 3′ss (Figure 2; Niemelä et al., 2015; Verbeeren et al., 2010, 2017; Olthof et al., 2021).

Currently, the only known cooperative interactions between the spliceosomes with demonstrated regulatory function have been reported for the *SNRNP48* and *RNPC3* genes. These encode the U11-48K and U11/U12-65K proteins, respectively, that are components of the U11/U12 di-snRNP. Both genes contain a conserved sequence element in non-coding regions which include a tandem repeat of the U12-type 5′ss consensus sequence. The tandem 5′ss sequences are recognized by the U11 snRNP, but are not used as splicing donors by the minor spliceosome. Instead, the binding of the U11/U12 di-snRNP activates an upstream U2-type 3′ss (Figure 2). Thus, the element has been aptly named a U11 snRNP-binding splicing enhancer, or USSE (Verbeeren et al., 2010). Interestingly, not only is the whole sequence stretch between the upstream 3′ss and the USSE element highly conserved, but the distance between the two sites also appears to be under evolutionary pressure to maintain optimal exon definition interactions between the two spliceosomes (Niemelä et al., 2015). These regulatory circuits function as autoregulatory or cross regulatory feedback mechanisms for both genes, promoting the formation of unproductive mRNA isoforms that are either degraded by NMD machinery (*SNRNP48*) due to inclusion of a PTC, or are retained in the nucleus due to an export-incompetent mRNA isoform (*RNPC3*) (Figure 2; Verbeeren et al., 2010, 2017; Niemelä et al., 2015). Notably, in *RNPC3* the same autoregulatory mechanism is also dynamically regulated during neuronal differentiation (Verbeeren et al., 2017), and is thus reminiscent of the post-transcriptional regulatory programs involving microexons and other RBPs (Ustianenko et al., 2017; Müller-mcnicoll et al., 2019).

#### Impact of Splicing Diseases on Spliceosome Cooperation

Further evidence for cooperative interactions between the two spliceosomes has emerged from global transcriptome analyses of diseases which partially compromise the function of the minor spliceosome. A typical outcome in these diseases is intron retention resulting from splicing defects. However, several studies have also demonstrated that the splicing defects are not limited to U12-type introns but can also spread to the flanking U2-type introns (Argente et al., 2014; Madan et al., 2015; Cologne et al., 2019; de Wolf et al., 2021; Olthof et al., 2021) in a subset of mRNAs. The most plausible explanation for these observations is that splicing of the affected U2-type introns is dependent on stabilizing exon-definition interactions with the neighboring U12-type introns (Figure 1B). Currently, evidence supporting this outcome is still somewhat limited and systematic surveys or more detailed mechanistic studies are needed to confirm these possibilities. Furthermore, there is currently no evidence of the regulatory significance of such interactions and it is possible that these interactions rather serve to reinforce constitutive splicing patterns.

### Competition Between Minor and Major Spliceosomes

**FIGURE 3:**
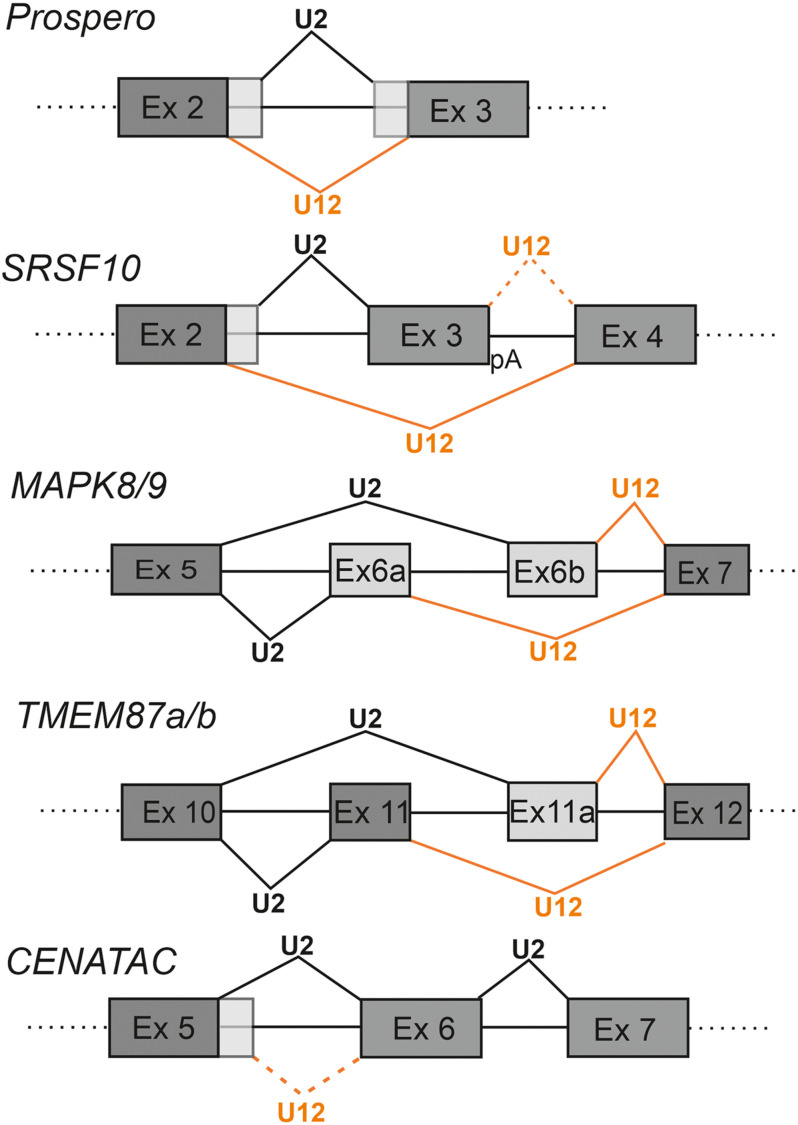
Competition between minor and major spliceosomes. Examples of known genes utilizing adjacent U12-and U2-type introns in regulating gene expression. The U2-type splicing is indicated with black lines and U12-type splicing with orange lines. Alternative exon sequences are indicated with light gray shading. With *SRSF10* an alternative poly-A site (pA) downstream of exon 3 is indicated. In the same panel, the rare U12-type splice site usage downstream of the exon 3 is indicated by dotted orange line.

Competition between the two spliceosomes represents a special subclass of alternative splicing where an individual intron can be spliced by either the minor or the major spliceosome. In these instances, competing splice sites are typically in close proximity to one another on the pre-mRNA and the resulting mRNA isoforms are also often annotated in public databases. Depending on the positioning of the splice sites, such introns have been referred to as twintrons or nested introns, which both refer to instances where either minor or major intron is located within the other intron type (Levine and Durbin, 2001; Scamborova et al., 2004; Chang et al., 2007; Hafez and Hausner, 2015). Nested U2-type and U12-type introns can either have separate 5′ss and 3′ss sequences or they can share one splice site, but not both. Another possibility is that the two introns are interlocked and have a partially overlapping configuration (Figure 3). The exact frequency and functional significance of these juxtaposed U2-type and U12-type splice sites in the genomic context has not been systematically characterized. Furthermore, it is likely that at least a subset of such events may have been annotated as standard alternative U2-type splice sites utilized by the major spliceosome (Levine and Durbin, 2001; Chang et al., 2007), particularly with the GT-AG subclass of U12-type introns, that can be misannotated as major introns. A small subset of these competition events involving adjacent U2-type and U12-type splice sites appear to have regulatory significance as suggested by Janice et al. (2013), who identified 18 twintron arrangements in the human genome that were evolutionarily conserved in vertebrates. An alternative hypothesis is that the nested introns may represent evolutionary intermediates in the process of minor to major intron conversion that has been suggested as an explanation for the low numbers of minor introns in present-day genomes (Burge et al., 1998; Mount et al., 2007; Lin et al., 2010; Janice et al., 2013; Moyer et al., 2020).

The first known and best characterized case of nested U12-type and U2-type introns has been described for the *prospero* (*pro*) gene in *Drosophila* (Otake et al., 2002; Scamborova et al., 2004). In this case the U2-type intron is located inside of an AT-AC subtype U12-type intron, resulting in either shorter (*pro-S;* minor spliceosome) or longer (*pro-L;* major spliceosome) mRNA isoforms. The resulting *pro* protein isoforms differ in the homeodomain region, which may affect DNA binding specificity (Figure 3). The balance between the two isoforms is developmentally regulated *via* a purine-rich enhancer element that binds the *Drosophila* hnRNPA1 homologs, Hrp36/Hrp38 (Scamborova et al., 2004; Borah et al., 2009).

A recent study on the *SRSF10* gene, a member of SR family splicing regulators, illustrates the use of competing nested U12-type and U2-type introns in regulating not only the levels of the SRSF10 protein, but also other members of the same family (Meinke et al., 2020). The *SRSF10* regulatory module resembles that of the *prospero* circuit as it contains a U2-type intron embedded within a U12-type intron with AT-AC termini (Figure 3). Splicing through the minor pathway leads to skipping of exon 3 and formation of the full-length *SRSF10* mRNA. In contrast, use of the major pathway leads to inclusion of exon 3 and formation of a truncated mRNA that utilizes a polyadenylation signal in exon 3. Competitive recognition by either the major or minor spliceosome, thus determines inclusion or exclusion of exon 3, which also harbors an exonic splicing enhancer (ESE) that is specific for the SRSFR10 protein and is involved in SRSF10 autoregulation. Levels of the functional *SRSF10* isoform spliced by the minor spliceosome correlate not only with the activity of the minor spliceosome, but also with overall levels of other SR proteins, in a tissue-specific and developmental manner. This further suggests that regulation of the SR-protein family as a group, may be linked to the activity of the minor spliceosome (Meinke et al., 2020).

Other known cases of nested introns with an external U12-type intron and internal U2-type intron have been described for the *HNRNPLL*, *ZNF207*, and *C1orf112* genes (Janice et al., 2013) but a clear regulatory role (if any) for these splicing events has not yet been determined. Similarly, instances of the reverse arrangement of nested introns in which the U2-type intron is located externally and the U12-type intron internally, have been reported in *NCBP2, PRMT1, dZRSR2/Urp, CTNNBL1, CUL4A*, and *SPAG16* genes (Lin et al., 2010; Janice et al., 2013). Of these, *NCBP2*, a subunit of the nuclear cap binding complex, represents a well-characterized regulatory switch where use of the major splicing pathway results in a truncated protein that lacks a large part of the RNA Recognition Motif (RRM) (Pabis et al., 2010). The truncated NCBP2 form does not support heterodimer formation with the other subunit (NCBP1) or cap binding, but instead has independent roles in transcription and RNA processing (Pabis et al., 2010).

Examples of more complex arrangements in which U12-type and U2-type introns are found in an interlocked and partially overlapping arrangement, have been described for the c-Jun N-terminal Kinase (JNK) family genes (*MAPK8-9*) and for *TMEM87a* and *TMEM87b* genes. Here, competition between minor and major pathways leads to mutually exclusive incorporation of alternatively spliced exons into the mRNA. In the *MAPK8*/*9* genes, U12-type and U2-type splice sites have an interlocked configuration where mutual competition of two U2-type 3′ss and two U12-type 5′ss leads to inclusion of either the alternative exon 6a or exon 6b (exons 7a and 7b in later genome assemblies, respectively) (Chang et al., 2007). In this case competition involves use of either the U2-type 3′ss upstream of exon 6b or the U12-type 5′ss downstream of exon 6a (Figure 3). The different JNK isoforms exhibit tissue-specific expression, such that the exon 6a isoform is predominantly expressed in neurons owing to the activity of neuronal splicing regulators, such as Nova (Relógio et al., 2005), whereas the isoform containing exon 6b is expressed ubiquitously (Chang et al., 2007). A recent study by Olthof et al. (2019) identified comparable examples in other MIGs, including the *TMEM87a* and *TMEM87b* genes. Similar to the *MAPK* circuit, both *TMEM87a and TMEM87b* contain an upstream U2-type intron with a 3′ss embedded in the downstream U12-type intron (Figure 3). Competition between the U2-type 3′ss upstream of exon 11a and the U12-type 5′ss downstream of exon 11 results in inclusion of either alternatively spliced exon 11 or exon 11a. Akin to the *MAPK* family genes, the different isoforms of *TMEM87a* and *TMEM87b* exhibit tissue-specific expression (Olthof et al., 2019), indicating that additional yet-to-be characterized regulatory factors play a role in splicing pattern selection in different tissues. Furthermore, the configuration of mutually exclusive alternative exons suggests that for both *MAPK8/9* and *TMEM 87a/b* genes, the regulation is linked to exon definition interactions between the minor and major spliceosomes across exons 6a/6b and 11/11a, respectively.

**FIGURE 4:**
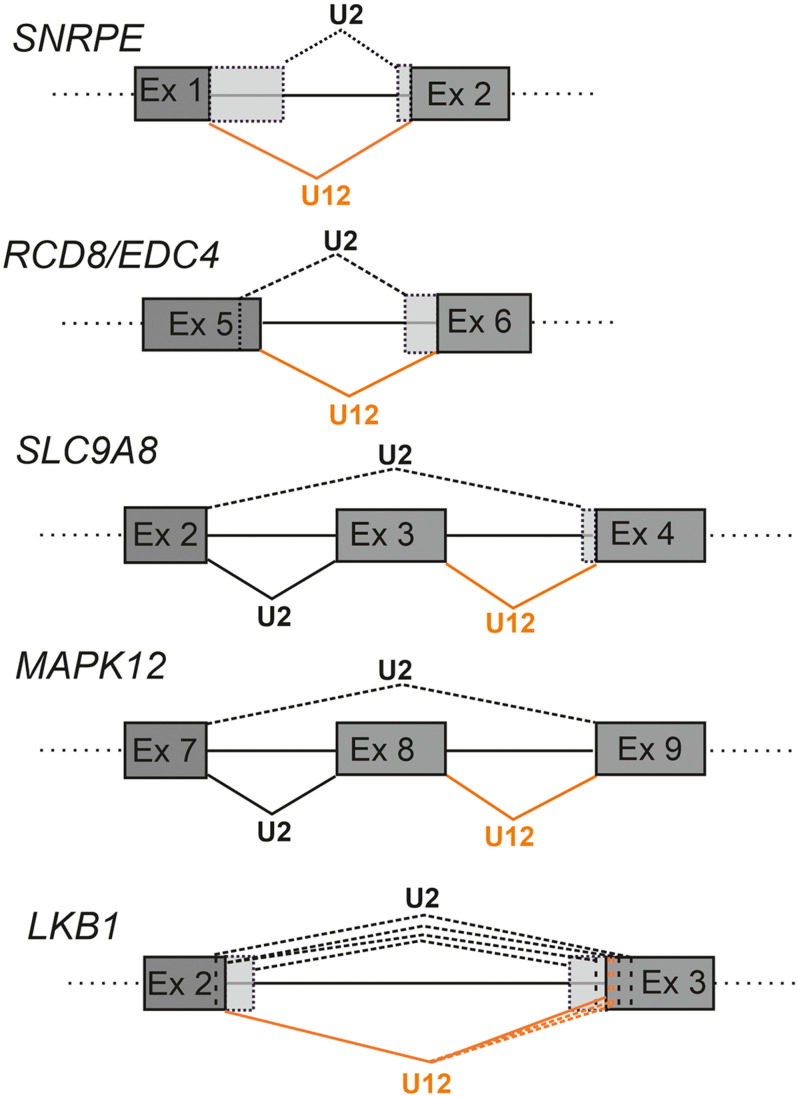
Schematics showing examples of cryptic splice site activation in defective minor intron splicing. The U2-type splicing is indicated with black lines and U12-type splicing with orange lines. Constitutive splicing is indicated with solid lines and cryptic splicing with dotted lines. Alternative exon sequences are indicated with light gray shading. With LKB1 the additional cryptic splice sites are indicated by dotted vertical lines.

Typically, competing U12-type and U2-type introns harbor distinct splice sites for either splicing pathway. However, in rare cases, one of the splice sites, usually the 3′ss, can be shared between the two splicesosomes. One such case has been described in the transcriptomic analysis of patients suffering from Microcephalic Osteodysplastic Primordial Dwarfism type I/Taybi-Linder Syndrome (MOPD 1/TALS) by Cologne et al. (2019). The study described an alternative splicing switch in patient cells from U12- to U2-type 5′ss usage within intron 5 of the *CCDC84* gene (later renamed *CENATAC*; de Wolf et al., 2021), while the 3′ss was shared between the two intron types (Figure 3). Splicing by the major spliceosome is expected to increase levels of the CENATAC protein since use of the minor pathway leads to incorporation of a PTC, and possibly a decay of the target mRNA by the NMD pathway. Interestingly, the CENATAC protein has recently been identified as a novel component of the minor spliceosome and particularly necessary for splicing of the AT-AN subtype of U12-type introns (de Wolf et al., 2021). Both the U12-type and U2-type 5′ss sequences are phylogenetically highly conserved, suggesting that the competing 5′ss elements are part of an autoregulatory feedback mechanism regulating the cellular levels of the CENATAC protein. A similar case has been observed for the *MAPK12* gene in a cell line carrying a U12 snRNA mutation linked to cerebellar ataxia. In that case minor spliceosome dysfunction induces an exon skipping event where the U2-type 5′ss of the upstream intron is used together with the 3′ss of the downstream U12-type intron (Norppa and Frilander, 2021; Figure 4).

In both the *MAPK12* and *CENATAC* cases with a shared 3′ss, there is another, albeit undetectable functional change upstream of the 3′ss. In the major pathway this region constitutes a pyrimidine-rich PPT recognized by the U2AF2 protein present in the U2AF1/2 heterodimer. In contrast, the minor spliceosome does not use the PPT or the U2AF1/2 heterodimer for intron recognition, but rather relies on BPS recognition by the U12 snRNA. A plausible explanation for the intron 3′ end compatibility between the two spliceosomes is that the minor intron BPS is also pyrimidine-rich and can, in a suitable context, also serve as a PPT for major introns, as suggested earlier (Burge et al., 1998).

A unique case of competition has been described for the non-productive use of components of both splicing pathways to regulate the ratio between unspliced genomic RNA and subgenomic spliced mRNA species in the Rous Sarcoma Virus (RSV). The genomic RNA of this retrovirus contains an inhibitory splicing element designated the Negative Regulator of Splicing (NRS), the function of which is to suppress all splicing of the viral RNA to ensure production of unprocessed genomic RNA, that is subsequently packaged into virions (Gontarek et al., 1993). Interestingly, the NRS contains overlapping binding sites for both U1 and U11 snRNPs (Gontarek et al., 1993; Hibbert et al., 1999). Investigations into the NRS function revealed that the binding of U1 snRNP is essential for splicing inhibition through non-productive interactions between U1 snRNP and with the factors bound to the downstream 3′ss (Cook and McNally, 1999; Hibbert et al., 1999). In this process the U11 snRNP is a competitive antagonist for U1 binding, thereby fine-tuning the NRS activity (McNally et al., 2004, 2006).

#### Impact of Splicing Diseases on Spliceosome Competition

Human diseases that compromise functions of either spliceosome can have two differential outcomes in competitive contexts between the two spliceosomes. First, in cases of competitive intron recognition that leads to a balanced expression of spliceosome-specific mRNA isoforms, such as those described above, the most likely outcome is a shift in the balance between the isoforms. This effect may vary depending on the specific mutation/spliceosomal defect, and may be combined with other outcomes, such as alterations in exon definition mechanisms or increased levels of intron retention.

An alternative possibility is the activation of cryptic splice sites near introns that do not display any obvious competition between the two spliceosomes. “Cryptic” in this context refers to splice sites that are not used under physiological conditions and are predictably also not annotated or documented in public databases. Thus, cryptic splice sites can be thought of as pseudo splices sites that are weaker in strength compared to authentic sites, and as such, are not efficiently recognized by the spliceosome in a context specific manner. Such splice sites tend to be activated as a consequence of mutations in either authentic splice sites or alternatively, in spliceosome components or regulators. Both lead to defects in splice site recognition either at the level of single introns, or more broadly (Kapustin et al., 2011). While cryptic splice sites may often lead to context-specific alternative splicing, mutations can also inadvertently generate new splice site sequences that closely match consensus splice site sequences and thus result in disease-specific alternative splicing of transcripts (Buratti et al., 2007). In essence, under physiological conditions the level of competition between cryptic splice sites and authentic sites is low, and the use of cryptic sites only becomes visible in disease contexts (Kapustin et al., 2011; Jaganathan et al., 2019). In more benign settings, cryptic splice sites are thought to give rise to tissue-specific alternative splicing, creating splice isoforms with diverse functions in different tissues (Jaganathan et al., 2019).

The consequences of competitive intron recognition by either minor and major spliceosome on cryptic splicing have thus far been studied in cases where the splicing of U12-type introns has been compromised (Turunen et al., 2008; Cologne et al., 2019; de Wolf et al., 2021; Norppa and Frilander, 2021; Olthof et al., 2021). In such cases, the outcome is typically increased minor intron retention combined with the activation of nearby cryptic U2-type splice sites. In more rare cases, splicing of the U12-type intron in question appears to be unperturbed and the splicing defect is observed only due to activation of U2-type cryptic splice sites such as those described for the *SNRNPE*, *RCD8/EDC4* and *SLC9A8* genes (Figure 4; Turunen et al., 2008; de Wolf et al., 2021; Norppa and Frilander, 2021).

Analysis of simple U12-type splice site mutations have been reported in a small number of detailed studies including the *LKB1* gene (also known as *STK11*) implicated in the Peutz-Jager Syndrome (Hastings et al., 2005) and the *WDFY1* gene (Chang et al., 2007). The *LKB1* case is particularly illuminating as the A > G mutation of the first nucleotide of the intron only changes the U12-type 5′ss subtype, with the U12-type 5′ss still matching the consensus sequence (Figure 4). Nevertheless, this leads to a complex pattern of cryptic splice site activation by both minor and major spliceosomes, suggesting that even small changes in splice site strength can tip the balance in the competition between authentic and cryptic splice sites (Figure 4).

Other genes in which splice site mutations have been shown to lead to activation of a U2-type cryptic splice sites include *SEDL*, which has been linked to spondyloepiphyseal dysplasia tarda (SEDT) (Shaw et al., 2003) and *AP4M1* that has been linked to Cerebral palsy (Verkerk et al., 2009) both of which exhibit activation of cryptic splice sites as a consequence of the mutation. More recently, an analysis of single nucleotide polymorphisms (SNPs) in U12-type introns and MIGs revealed that such variants have a much wider connection to disease than previously thought (Olthof et al., 2020). In contrast to these splice site point mutations, mutations in components of the minor spliceosomal machinery are characterized by high levels of intron retention and additionally, cryptic splice activation in a larger number of mRNAs as evidenced by several transcriptomic studies (Madan et al., 2015; Merico et al., 2015; Cologne et al., 2019; de Wolf et al., 2021). The studies described above provide evidence for complex cryptic splicing events that not only disturb the splicing of immediate surroundings of the affected U12-type introns, but also extend further and influence the splicing of more distal U2-type introns, possibly as a consequence of disrupting the exon definition networks.

## Discussion

Until very recently, the outlook on the regulatory significance of the minor spliceosome functioning in parallel with the major spliceosome has been static. The main focus in the field has been on the inefficient splicing of U12-type introns under physiological conditions and the increased intron retention events observed in minor spliceosome diseases. However, rapidly accumulating transcriptomic data from an increasing number of minor spliceosome diseases is now challenging this narrow view by providing robust evidence of widespread crosstalk mechanisms between the minor and major spliceosomes, which were previously only reported in single-gene investigations. The fact that the locations of U12-type introns are known and highly conserved, presents a unique opportunity or lens through which the parallel functioning of both spliceosomes, particularly in exon and intron definition contexts, can be examined. Importantly, under physiological conditions, crosstalk between the two spliceosomes appears mostly to function as a mechanism for reinforcing constitutive splicing patterns through exon definition interactions, and to a lesser extent, as a mechanism for regulating balanced expression of mRNA isoforms that are dependent on either spliceosome. The less studied role of this crosstalk in regulating gene expression is particularly intriguing, as the few genes described here that utilize adjacent U12-and U2-type splice sites to regulate their expression, highlight an overlooked yet significant regulatory mechanism for some MIGs. Consequently, examining such crosstalk mechanisms has the potential to contribute to current understanding of the evolutionary significance of both spliceosomes functioning in parallel. Interestingly, for genes currently known to harbor these adjacent splice sites both cooperative and competitive outcomes have been observed, with the latter being more common. Competition between splice sites is a common occurrence for U2-type introns, due to the more degenerate splice sites in these introns and has been discussed extensively within the context of alternative splicing (Chen and Manley, 2009; Wang et al., 2015; Dvinge, 2018; Ule and Blencowe, 2019). In contrast, competition between U12- and U2-type splices sites is relatively understudied and much remains to be understood regarding such competitive events, including the effects of enhancers and silencers. It would thus be interesting to determine if weaker U12-and U2-type splice sites are a common feature of nested introns and whether specific U12-type subtypes are preferred for these competitive events.

In disease contexts the primary splicing defect can lead to additional defects in both competitive and collaborative interactions between the two spliceosomes. The occurrence of additional mRNA isoforms as a consequence of the loss of crosstalk between the two spliceosomes also influences the interpretation of transcriptomic data derived from minor spliceosome disease patient cells. A typical transcriptome-level workflow aims to identify the affected minor introns and MIGs, and optionally, use the intron retention levels to estimate the downregulation of MIGs for downstream analyses. Thus, the presence of novel transcripts arising from cryptic splice site usage and the loss of exon definition interactions can either exacerbate the effect on expression levels or lead to formation of mutant proteins, which may contribute to the pathology of the given disease. Additionally, the *MAPK8/9* (Chang et al., 2007) and *TMEM87a/b* (Olthof et al., 2019) examples, as well as the MOPD1/TALS analyses (Cologne et al., 2019) have demonstrated that differential U12- or U2-type splice site usage is not static, and can change in a tissue-specific manner, necessitating extended analyses of multiple cells and tissue types. Such analyses may provide insight into the specificity that is observed in minor spliceosome diseases, in which developmental processes and neuronal tissues are particularly affected (Jutzi et al., 2018; Olthof et al., 2020).

At a more technical level, the activation and detection of cryptic splice sites poses an additional challenge for data analysis. Most software used in alternative splicing analyses rely on existing annotations when detecting alternative splicing events (Jiang and Chen, 2021). As cryptic splice sites are not normally annotated in public databases, they tend to be ignored by most alternative splicing analysis tools. This can be mitigated by using software that allows for the identification of *de novo* events, such as KisSplice used in the Cologne et al. (2019) study or MAJIQ which examines local splicing variation complexity (Vaquero-Garcia et al., 2016). In our recent work on Mosaic Variegated Aneuploidy (MVA) caused by mutations in *CENATAC* (de Wolf et al., 2021), we documented complex patterns of cryptic splice site activation as a consequence of minor intron splicing defects using Whippet (Sterne-Weiler et al., 2018), which is particularly suited for deciphering complex alternative splicing events. It is, however likely that in future, analyses of complex splicing events in transcripts may be resolved by the use of long-read sequencing methods that are less sensitive to annotation biases.

In summary, recent transcriptome-wide investigations have uncovered a substantial number of crosstalk interactions between the major and minor spliceosomes. An outstanding question related to these splicing events concerns the identification of true regulatory events from cases representing opportunistic cryptic splice site activations. An additional unanswered question is whether either spliceosome can be regulated individually in a manner that would influence the crosstalk. There is some evidence of specific splicing factors such as hnRNP H/F and SRSF10 being linked to regulation of the minor spliceosome (McNally et al., 2006; Turunen et al., 2013b; Meinke et al., 2020), but their generality remains to be confirmed. The currently known instances of cooperative and competitive interactions between the two spliceosomes highlighted in this review emphasize their functional and regulatory potential and set the stage for future investigations of their significance.

## Author Contributions

Both authors contributed equally to the article and approved the submitted version.

## Conflict of Interest

The authors declare that the research was conducted in the absence of any commercial or financial relationships that could be construed as a potential conflict of interest.
